# Rehabilitation for people living with dementia: A practical framework of positive support

**DOI:** 10.1371/journal.pmed.1002245

**Published:** 2017-03-07

**Authors:** Linda Clare

**Affiliations:** Centre for Research in Ageing and Cognitive Health, University of Exeter, Exeter, United Kingdom

## Abstract

In a Perspective, Linda Clare proposes using a cognitive rehabilitation approach for people living with dementia.

Awareness of the need to improve accessibility of services and opportunities for people with disabilities is growing, but people with “hidden” disabilities such as dementia can be excluded from these developments. Conceptualizing dementia in terms of social disability highlights the way in which symptoms such as memory problems—and the secondary effects of these, such as loss of confidence or negative reactions from others—affect the possibility of engaging in activities and participating in society [1]. It also suggests some practical solutions that can support participation and inclusion and promote the ability to live well [2]. Activity limitation and participation restriction can be tackled from two directions. From a community perspective, the focus is on dismantling external barriers to participation by changing public attitudes and creating accessible, dementia-friendly environments. A growing social movement led by people with dementia, Alzheimer associations, and supporters promotes acceptance, inclusion, and awareness of rights [3]. From a personal perspective, the focus is on enabling people with dementia to participate in everyday life, and in their families and communities, in a way that is meaningful to them. This is the aim of rehabilitation [4].

## Why is rehabilitation relevant?

The experience of disability confers a right to rehabilitation for people living with dementia. The United Nations Convention on the Rights of Persons with Disabilities outlines the right of people with disability to be able to attain and maintain maximum independence, with the assistance of comprehensive rehabilitation services (Article 26(1)) [5]. We are used to thinking of rehabilitation in terms of physical rehabilitation following injury, but it is equally relevant for people with cognitive, rather than physical, impairments. This includes people whose impairments result from long-term, progressive neurodegenerative conditions. The rehabilitation of people with cognitive impairments is called cognitive rehabilitation [6]. In community settings, this approach may be called reablement or restorative care, and from a public health perspective, it can be considered synonymous with tertiary prevention. These concepts share similar aims [7], and for convenience, I will use the term “rehabilitation” here. Rehabilitation provides both a set of guiding principles to shape a model of service provision and a coherent practical framework for supporting people with dementia and their families [8].

## How can cognitive rehabilitation benefit people with dementia and carers?

A rehabilitation-focused service would be organized around key principles of enabling people to function optimally in the context of their intrinsic capacity and current health state. This means ensuring that people are as independent as they wish to be, have as much control as possible over daily life, have opportunities to engage in meaningful roles and activities, and are able to integrate the changes they experience into a coherent and enduring sense of identity. The rehabilitation philosophy is genuinely person centred [9] and reflects important values underpinning good dementia care. Rehabilitation involves working with people to achieve the goals that are important to them. It is based on individual formulations and not a one-size-fits-all approach [10], acknowledging that each individual has a unique set of experiences, values, motivations, strengths, and needs.

In cognitive rehabilitation, these principles are applied to enable people with dementia to maintain or optimize functioning. The term “cognitive” is perhaps misleading, as cognitive rehabilitation does not set out to train or improve cognition but uses a goal-oriented approach to facilitate improved management of functional disability. Potential targets include everyday functioning, activities of daily living, self-care, language and communication, social interaction, and the effects of dementia-related physical disability. Cognitive rehabilitation therapists work collaboratively with each individual to formulate meaningful goals that are realistic and potentially achievable. They evaluate the person’s strengths and the resources needed for goal attainment, identify areas of mismatch, and collaboratively develop a plan to support goal attainment or address the identified need using evidence-based methods. These might involve new learning, relearning, use of compensatory strategies, or a combination of these. The person’s intrinsic capacity may be augmented by additional resources such as assistive technology. Therapists also provide important psychological support as people confront the emotional impact of functional disability.

Principles of rehabilitation can be flexibly applied to address different types of need at various stages of dementia. For example, a person in the early stages of dementia may want to learn to use email to keep in contact with friends, develop strategies to feel confident enough to go out alone, or be able to cook a meal without getting distracted, while for someone with more advanced dementia, the focus may be on maintaining the ability to dress herself, managing difficulties with swallowing, or enabling participation in an enjoyable activity. Each individual might have several episodes of rehabilitation support over time as needs and goals change or as particular circumstances arise, such as a return home after hospitalization. Cognitive rehabilitation is usually carried out in the setting where the person lives or undertakes activities, to ensure direct relevance, and carers and families, when available and willing, are fully involved and appropriately supported. There is a small but growing evidence base demonstrating that cognitive rehabilitation is effective in supporting everyday functioning, reducing disability, and delaying institutionalization [11–15].

## Where do other nonpharmacological interventions fit in?

Many different types of nonpharmacological intervention for people with dementia have been described. Most of these are not cognitive rehabilitation; it is important that this term is understood correctly and is not applied to interventions that do not warrant it. Some approaches, however, are directly complementary to cognitive rehabilitation. These either address related aims (for example, self-management groups to enhance self-efficacy or therapeutic groups to promote adjustment to living with dementia) or address problems that negatively affect functioning and participation (for example, psychotherapy for depression or individualized interventions for agitation). Also directly relevant is support for family carers. Other intervention approaches focusing on providing pleasurable and meaningful activity or encouraging social contact can complement rehabilitation by creating opportunities to enjoy positive experiences and relationships. Within a rehabilitation-focused service, an intervention pathway would include these approaches where there is evidence that they offer benefits. Less likely to be recommended in the context of a rehabilitation model are nonpharmacological interventions that address symptoms in isolation or out of context so that gains, if any, are unlikely to transfer to daily life. People with dementia should also have full access to specialist physical rehabilitation where needed following injury or illness, as well as any other appropriate medical treatment [16].

## How could services adopt a rehabilitation model?

A rehabilitation-based model of positive support could potentially be introduced in part through a redeployment of available resources. The costs of this positive approach might be offset to some extent by preventing difficulties, limiting the costs of managing distressing symptoms, and delaying institutionalisation. There is a need to develop service systems with a clear focus on optimizing functioning and supporting relationships, identity, and engagement and a need to train staff to implement rehabilitative interventions. It is essential to fully involve people with dementia and carers to ensure a thorough understanding of their perspectives.

## Why should we acknowledge the right to rehabilitation?

Acknowledging the right to rehabilitation offers a tremendous opportunity to create a focused and coherent approach to positive support for people with dementia, of any age, subtype, or severity, and their families. A rehabilitation model offers a guiding framework for services and for health and social care practitioners and a practical means of providing person-centred, evidence-based interventions to maintain or enhance functioning, engagement, and participation.
